# Validity of Estimated Results from a Wearable Device for the Tests Time Up and Go and Sit to Stand in Young Adults and in People with Chronic Diseases

**DOI:** 10.3390/s23125742

**Published:** 2023-06-20

**Authors:** Kokouvi Geovani Agbohessou, Stephanie Sahuguede, Justine Lacroix, Fadel Hamdan, Emmanuel Conchon, Yannick Dumas, Anne Julien-Vergonjanne, Stephane Mandigout

**Affiliations:** 1 HAVAE EA6310 (Handicap, Aging, Autonomy, Environment), University of Limoges, 87042 Limoges, France; 2 XLIM Laboratory, UMR CNRS 7252, University of Limoges, 87000 Limoges, France; 3 Développement de Logiciels, UNOVA, 87000 Limoges, France; 4 ILFOMER (Institut Limousin de Formation aux Métiers de la Réadaptation), Université de Limoges, 87000 Limoges, France

**Keywords:** accelerometer, gyrometer, wearable sensor, reliability, physical activity, chronic disease

## Abstract

Background: Health care professionals need a valid tool to assess the physical ability of patients with chronic diseases. We aimed to assess the validity of the results of physical fitness tests estimated by a wrist wearable device in young adults and chronic disease people. Methods: Participants wore a sensor placed on their wrist and performed two physical fitness tests (sit to stand (STS) and time up and go (TUG)). We checked the concordance of sensor-estimated results using Bland–Altman analysis, root-mean-square error, and intraclass coefficient of correlation (ICC). Results: In total, 31 young adults (groups A; median age = 25 ± 5 years) and 14 people with chronic diseases (groups B; median age = 70 ± 15 years) were included. Concordance was high for both STS (ICC_A_ = 0.95, and ICC_B_ = 0.90), and TUG (ICC_A_ = 0.75, ICC_B_ = 0.98). The best estimations were given by the sensor during STS tests in young adults (mean bias = 0.19 ± 2.69; *p* = 0.12) and chronic disease people (mean bias = −0.14 ± 3.09 s; *p* = 0.24). The sensor provided the largest estimation errors over 2 s during the TUG test in young adults. Conclusion: This study showed that the results provided by the sensor are consistent with those of the gold standard during STS and TUG in both healthy youth and people with chronic diseases.

## 1. Introduction

Chronic diseases have a significant impact on life expectancy and almost 20% of people have experienced these conditions in France [1]. Chronic diseases represent a major public health issue in France and the most common are cardiovascular diseases (5.1 million people treated in 2019), diabetes (4.0 million), chronic respiratory diseases (3.7 million), and cancers (3.3 million) [2].

In a recent systematic review, studies have mentioned the existence of a positive effect of the practice of physical activity (PA) on the health of these people [3]. Indeed, it has been observed that following PA programs improve the functional abilities of people with chronic diseases [4,5]. However, these programs can be difficult to access for patients, due to lack of availability on the scheduled sessions, lack of economic resources or significant geographical distance. Limited adherence to these PA programs is generally observed in these patients [6]. Concern about the lack of coordination and continuity of care for chronic illness has led to a series of reforms in the health care field. The development of telemedicine in France has encouraged the remote management, evaluation and monitoring of patients with chronic diseases [2].

Monitoring PA levels, to guide rehabilitation, is a challenge for clinicians. Personal activity monitors are becoming increasingly popular and offer the potential to improve rehabilitation protocols at a distance. Measuring its own PA performance is important for adults with chronic diseases to comply with PA recommendations in clinical practice [7,8].

The recent development of small, wearable electromechanical sensors may bridge the gap between the practicality of commonly used clinical tools and more accurate, but expensive and difficult-to-access technological systems for patients. This reduced complexity may encourage more clinicians who are unfamiliar with the technical aspects of the new equipment to integrate sensors into practice [9]. 

Among these sensors, there are the inertial motion units (IMU) that encompass accelerometer, magnetometer and gyroscope axis. In a five-year update, Picerno et al. showed that IMU, when combined with data processing software, can define person movement, heading and orientation [10]. The accuracy of wearable IMU has improved over time with several studies validating its use for PA measurement such as step count, walked distance, speed measurement and energy expenditure in free-living conditions [10,11,12].

In clinical practice, health professionals used valid physical fitness tests to assess chronic disease patients. These tests are quick, require no special device or training, and are easily included as part of the routine medical examination. Among the tests commonly used by professionals, sit to stand test (STS) assesses lower body strength, needed for daily life activities. It (the number of STS completed) has shown a good correlation (r = 0.75) with the gold standard (dynamometer) in older people [13]. On the other hand, the timed up and go test (TUG) assesses the functional mobility and dynamic balance of persons and allows the detection of older adults at risk of falling [14]. The TUG has also excellent test-retest reliability (ICC = 0.95) for individuals with chronic stroke for example [15].

In contrast to healthy subjects, in chronic disease patients, functional heterogeneity such as slowness of the movements, abnormalities in gait, heart rate and metabolism make the assessment of PA particularly challenging using wearable IMU. Research on the validity and reliability of these devices in people with chronic diseases is limited [16]. Above all, it is also important to validate the device in a population of young healthy adults in order to optimize its functioning.

The main aim of the chronic patient rehabilitation process is to optimize patients’ performances of motor tasks in daily life. Therefore, clinicians need a reliable and valid measurement that will allow them to establish the patient’s functional mobility and monitor the patient’s progress at a distance [17].

Previous works using combined data acquired from multiple device placements (e.g., devices placed at hip and trunk, smartphone-based or ultrasound-based sensor) showed that devices could be used to study transitions in STS test [18] and kinematic parameters in TUG tests [19]. These wearable devices require multiple sensors and the sensor to be placed on the L3 region of the subject’s lumbar spine [18,19]. Wear several accelerometer devices slightly enhance activity recognition accuracy in chronic disease patients. However, given the extra burden of wearing additional devices for patients, single accelerometers with appropriate placement appear to be sufficient for estimating the number of STS and TUG activity times in chronic disease patients [20,21]. The use of an IMU, therefore, appears to be sufficient to determine people’s level of physical fitness through the STS and TUG. To our knowledge, no study has evaluated the accuracy of a simple wrist wearable IMUs sensor for performing these clinical tests.

Most of the existing sensors are closed to reprogramming; we have therefore developed a personal sensor that has allowed us to implement our algorithm capable of evaluating the muscular strength, and dynamic balance of chronic disease people.

The purpose of this study was therefore to evaluate the validity and internal reliability of STS and TUG tests estimated by our personal custom device in young adults and people with chronic diseases. 

## 2. Materials and Methods

### 2.1. Participants Selection

We have carried out a cross sectional exploratory study. Volunteer participants were allocated to two groups; group A (healthy young adults aged from 18 to 35 years old) and group B (adults with chronic diseases of more than 6 months duration). The healthy group was the test group and consisted of student volunteers who met our inclusion criteria and reported no chronic diseases. For group B (Who had diseases such as diabetes, breast cancer, multiple sclerosis, morbid obesity...), chronic disease participants were contacted through patient associations. We have considered several pathologies to reflect the variability of illnesses among people with chronic diseases.

They were recruited in a laboratory setting and all tests were performed in a gymnasium. Participants with acute cardiac or respiratory pathologies were not included. Participants who had any conditions that might affect the assessment protocol, such as neurological or musculoskeletal problems affecting mobility and hindering proper assessment were not included. The therapist responsible for the protocol informed the participants of the details of the protocol and registered their consent. The research protocol was approved by an ethics committee, “Comité d’Ethique pour la Recherche en STAPS” (CERSTAPS, Notice number: IRB00012476-2023-16-01-220). 

### 2.2. Instrumentation

#### 2.2.1. IMU-Based Custom Device Description 

**Figure 1:**
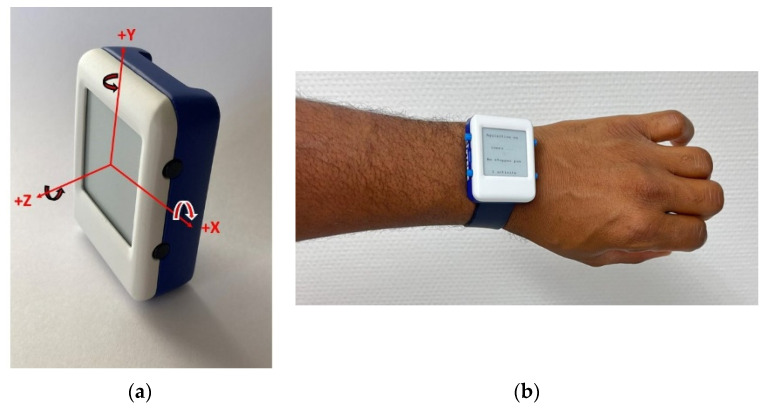
Orientation of the axes and device placement. (**a**) Orientation of the axes. (**b**) Device placement and stand-by screen.

We developed a custom wearable system able to be worn at the wrist (Figure 1), based on the ICM20948 9-axis IMU from STMicroelectronics. The system includes a processing unit (STM32WB55RG) for acquisition, storage and communication, a memory unit, a communication unit based on both USB and Bluetooth connectivity, and a screen and buttons to interact with the user. In order to deal with memory size and consumption, the data from the IMU are stored in the device and transferred by USB at the end of the exercises. The sampling frequency is set to 50Hz in order to be able to memorize values from each of the 9 axes. We performed the same test with the two sampling frequencies of 50 Hz and 100 Hz. It was verified that the results were identical.

Figure 1a shows the orientation of the axes on the prototype and Table 1 shows the main characteristics of the device.

The data are then post processed on a computer unit, in order to determine the duration of STS and TUG exercises.

#### 2.2.2. The Algorithms

We processed several sets of values in order to identify among all recorded data (accelerometer, gyrometer and magnetomer, each over 3 axes) which are the most suitable for the analysis of the tests (number of sit-to-stand performed during STS test or time duration of TUG test). The algorithms have been set up following an iterative methodology: a preliminary version based on the analysis of the signal of some healthy subjects has been established. Then, we adapted them with the analysis of the signals corresponding to a group of people with chronic diseases. Each algorithm methodology is explained in detail in the following paragraphs.

##### STS Algorithm

The objective of the processing is to determine the number of sit-to-stand completed in 30 s from the raw data of the recorded values from the IMU.

First, we observed based on preliminary tests that the accelerometer data on each of the 3 axes gives interesting information compared to the other raw data and that it is possible to observe periodic variations linked to the repetitiveness of the exercise. The analysis of a large set of data permitted us to identify in particular that the x-axis of the accelerometer raw data was the one from which information could be extracted the most reliably. Periodic spikes permit identifying the participants’ successive chair lifts during the test.

From these raw data, the first processing consisted in carrying out low-pass filtering in order to smooth the values and remove insignificant variations. We used a 4th order Butterworth filter with a cutoff frequency of 0.5 Hz. Indeed, this low value is justified by the fact that it is not possible to perform more than two chair lifts in 1 s.

To determine the number of chair lifts, the strategy then adopted is to count the number of points passing through the median of the curve. 

However, for chronic disease, people slow down the speed at which they perform the test because of their diseases, the tracings become irregular and compared to a simple median introduces counting errors. To solve this problem, a sliding median was calculated every 2 s.

**Figure 2:**
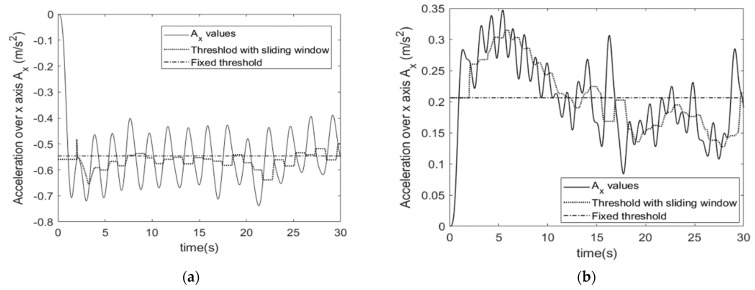
Illustration of sliding window process on x-axis if accelerometer data to identify spikes in the sit to stand (STS) algorithm. (**a**) Example issued from heathy group. (**b**) Example issued from chronic disease group.

Figure 2 shows an example of a plot with a fixed threshold and with a sliding window, for two different persons from the healthy group (Figure 2a) and from the chronic disease one (Figure 2b). These curves highlight the fact that considering a fixed threshold may introduce errors in the counting. Even for a healthy person having regular exercise, some spikes are not identified: in the example (Figure 2a), only one at the beginning of the exercise is missing. In the case where the exercise realization is not regular such as in Figure 2b, it conduces to totally erroneous values. The sliding window with a 2 s width undercomes this issue.

##### TUG Algorithm

In this case, the objective is to determine the time taken by the participant to perform the TUG test. It is supposed to be lower than 30 s.

Preliminary tests permitted us to identify that the most significant data to be analyzed during this test is the angular velocity data from the gyrometer, recorded for 30 s, and more precisely the z-axis of gyrometer. In order to remove insignificant variation, the first processing is low-pass filtering as in the STS test. 

We used a 4th order Butterworth filter with a cutoff frequency of 5 Hz. Indeed, we have empirically determined from the different recorded sets of values, that the cutoff frequency should be between 4 Hz and 7 Hz. A higher cutting frequency leads to insignificant perturbations in the signal, whereas a lower removes useful data. The efficiency of this cutoff frequency has been verified for people having a mean speed of walking around 0.5–1.5 m/s depending on people’s health condition and age.

**Figure 3:**
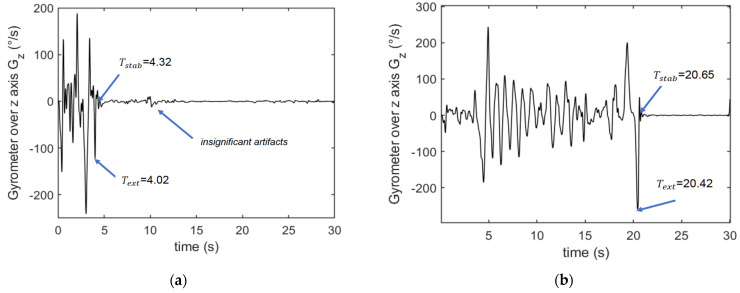
Illustration of z-axis of gyrometer evolution of timed up and go (TUG) exercise. (**a**) Example issued from heathy group. (**b**) Example issued from chronic disease group.

Figure 3 shows two examples of z-axis gyrometer data recording during the TUG for two very different profiles. The objective is to evaluate the duration of the test $T_{TUG}$ which is between the last extremum on the plot and the interval where the angular velocity is stable, corresponding to the phase where the participant has returned to sit down on the chair and where he is at rest.

The method used then consists in identifying on the recording, the instant at which stability is reached, denoted as $T_{stab}$ and extracting the time $T_{ext}$ where the plot exhibits a local extremum. The end of the test is between these two values $T_{ext}$ and $T_{stab}$.

Empirically we considered that the duration of the test is equal to:

(1) $$T_{TUG}=\frac{\left(T_{stab}+T_{ext}\right)}{2}$$

In order to remove artifacts of movements, we determined that the stability instant corresponds to the time at which the variations in angular velocity are less than 10°/s during a duration of 3 s. The points corresponding to $T_{ext}$ and $T_{stab}$ are pointed out in the plot’s examples in Figure 3. 

### 2.3. Physical Fitness Tests

We have selected a gold standard for each test that has been validated in chronic disease people.

#### 2.3.1. Sit to Stand Test

The STS aimed to assess lower body strength [22]. Subjects were instructed to stand up fully and sit down in a chair with their back against the rest as fast as possible in 30 s with their arms crossed over their chest throughout. The number of repetitions performed during the 30-s STS test was simultaneously recorded by the examiner (gold standard) and our custom device [13]. STS performance is known to be associated with disability, falls, hip fracture and mortality among older adults and chronic disease people [23].

#### 2.3.2. Timed “Up and Go” Test

The TUG assesses the functional mobility of persons. The subject stands up from a chair, walks 3 m forward, turns around, walks back and sits down again [14]. The end of the walkway was clearly marked with a pin. Subjects were instructed to sit straight on the chair with their hands on their thighs and their backs touching the back of the chair. After the tester gave them the go signal, they arose from the chair, walked at their normal speed, turned around right after passing the pin at the end of the pathway, returned back to the chair, turned around and sat down. The time to complete the test is recorded on the one hand by the investigator using a Handheld Stopwatch (HHS) and on the other hand, is estimated by the custom device.

### 2.4. Test Protocol and Data Processing

Data collection took place in France between September 2022 and November 2022. Self-reported measures were given by participants. Body mass index (BMI) was calculated from these measures. Participants were advised to wear suitable sports clothing and footwear for the test.

**Figure 4:**
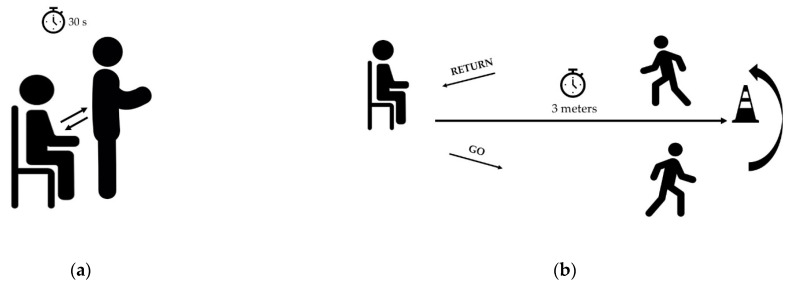
Illustration of the test protocol. (**a**) Sit to stand (STS) test. (**b**) Timed up and go (TUG) test.

The test protocol involved the following steps:

A. Warm-up session with stretching, joint mobilization and muscle strengthening exercises.
B. Installation of the custom device placed on the left wrist of the participant during all tests. The Installation of the sensor required a maximum of 3 min. Prior to use by each participant the device was automatically initialized via the supporting software (USB, prototype). To perform a test, the investigator had to choose the test from the menu, press the “start” button and 3 s later the device vibrated and the participant could start the test, see Figure 1, Figure 2 and Figure 3.
C. Prior to each test, the participant performed a trial run to ensure that they understood the instructions (this step may take about 2 min). The participants performed STS and TUG tests, the results were also measured by the examiner using visual counts for STS and a stop-watch for TUG. The activity mode was automatically disabled at the end of each test. The data collection is carried out during all the tests which is supposed to take no more than 2 times 30 s, so less than 1 min. With the given acquisition frequency of 50 Hz, this corresponds to 3000 measurements. Each measurement corresponds to 9 values issued from the 9-axis accelerometer. With the 12 bits-ADC of the device, a 1-min recording corresponds to less than 40 kbytes.
D. Download of the data from the device: Data from the sensor was downloaded via a universal serial bus (USB) and then processed. The members of the research team used a laptop to configure the device, start a test, and read the outcomes at the end of each test. The testing procedure is illustrated in Figure 4.

### 2.5. Statistical Analysis

Data analysis was performed with R version 4.2.1. The normality of the distribution of data was examined using the Kolmogorov-Smirnov test [24,25].

Descriptive statistics were calculated for all participants’ characteristics. Variables will be expressed as median with interquartile range [IQR], mean ± standard deviation (SD) or number (percentage), according to the normality of the distribution of data.

Sample size numbers were determined by procedures described by Walter et al., for inter-device reliability [26]. We calculated the required sample size based on a previous study [7]. Twenty-five subjects were deemed to be acceptable to judge the difference between two devices with a minimally acceptable level of 0.5, when α = 0.05 and β = 0.20 (power = 0.8). The risk of data loss related to possible malfunctioning devices was also included in the calculation. We estimated this risk at about 15%, so we needed a total of 30 individuals.

Validity was evaluated for the personal sensor estimations and the measures observed by the investigator during every test. We used the Spearman correlation [27] to determine if both methods of measurement analysis produced comparable results. 

Intraclass Correlation Coefficients (ICC) [28] were used for the inter-rater analysis to determine if the custom device can be used with confidence and reliability to assess physical fitness in people. Reliability and validity were assessed using the criteria suggested by Terwee et al. [29]. Based on the 95% confidence interval of the ICC estimate, values less than 0.5, between 0.5 and 0.75, between 0.75 and 0.9, and greater than 0.90 are indicative of poor, moderate, good, and excellent reliability, respectively.

Inter-rater reliability was visualized using Bland–Altman plots with a mean bias (MB), 95% limits of agreement (LoA) calculated using the formula: 95% LoA = mean difference ± 1.96 × SD [30]. We also appreciated differences between values given by the custom device and the values observed by the investigator using the root-mean-square error (RMSE).

There is currently no conventional cutoff in the literature to define the acceptable levels of inter-observer agreement. A minimum possible error is expected. Based on prior studies and clinical experience, any differences between raters greater than ±5 units were considered clinically significant, and exceeding ± 10 units were considered unacceptable [31]. The threshold of significance for all tests was 0.05 and 95% confidence intervals (CI) were reported.

## 3. Results

### 3.1. Population Characteristics 

Fourteen people with a chronic disease and thirty-one healthy young adults were recruited in this study. The median age was 70 [15] years in the chronic disease group and 25 [5] years in the healthy group. The proportion of females was higher in the chronic disease group than in the healthy group (64% vs. 48%). The average BMI was significantly higher in the chronic disease group than in the healthy group (31.7 ± 3.6 vs. 24.6 ± 3.9) Kg·m^−2^. In both groups, the average number of STSs with the gold standard was the same as with the customized device.

In addition, in the healthy group, the average number of seconds in the TUG test with the gold standard was slightly higher in the healthy group (5.9 ± 0.8 vs. 5.5 ± 1.2 s), but in the chronic disease group, the gold standard showed lower seconds in the TUG test than the custom device (6.7 ± 3.1 vs. 7.0 ± 3.4 s). Descriptive statistics for all the subjects are presented in Table 2.

### 3.2. Validity Parameters

In young healthy adults (group A), TUG showed a significant (*p* < 0.001) and good correlation (r = 0.80) between the custom device and investigator measures. STS test in the healthy adults’ group demonstrated also a significative correlation (r = 0.96, *p* < 0.001). The TUG test showed less reliability in the heathy adults (ICC_A_ = 0.75, *p* < 0.001) than in chronic disease participants (ICC_B_ = 0.98, *p* < 0.001). Reliability was high for the STS test, in young adults (ICC_A_ = 0.95, *p* < 0.001) and chronic disease participants (ICC_B_ = 0.90, *p* < 0.001). Both, TUG (r = 0.87, *p* < 0.001) and STS (r = 0.94, *p* < 0.001) tests in chronic disease participants (group B) showed a significative correlation. The details of the correlations are summarized in Table 3. 

The parameters of validity of each method are presented in Table 3. In the chronic disease group, the custom device slightly overestimated the number of trials performed for STS (Mean Bias (MB) = −0.14) and the number of seconds performed on the TUG test (MB= −0.36). The best estimations were given by the sensor during STS tests in chronic disease patients (MB = −0.14 s; *p* = 0.24) and in young adults (MB = 0.19; *p* = 0.12). The large estimation errors over 2 s (*p* < 0.05) were provided by the custom device during the TUG test in young adults (MB = 0.45; 9.24%).

On the other hand, the most accurate estimations were obtained using the custom device for STS tests in young adults (RMSE = 7.22%), chronic disease people (RMSE = 10.83%) but also for the TUG test in chronic disease people (RMSE = 11.02%). The custom device showed lower accuracy for the TUG test in healthy adults (RMSE = 15.75%), (Table 4).

**Figure 5:**
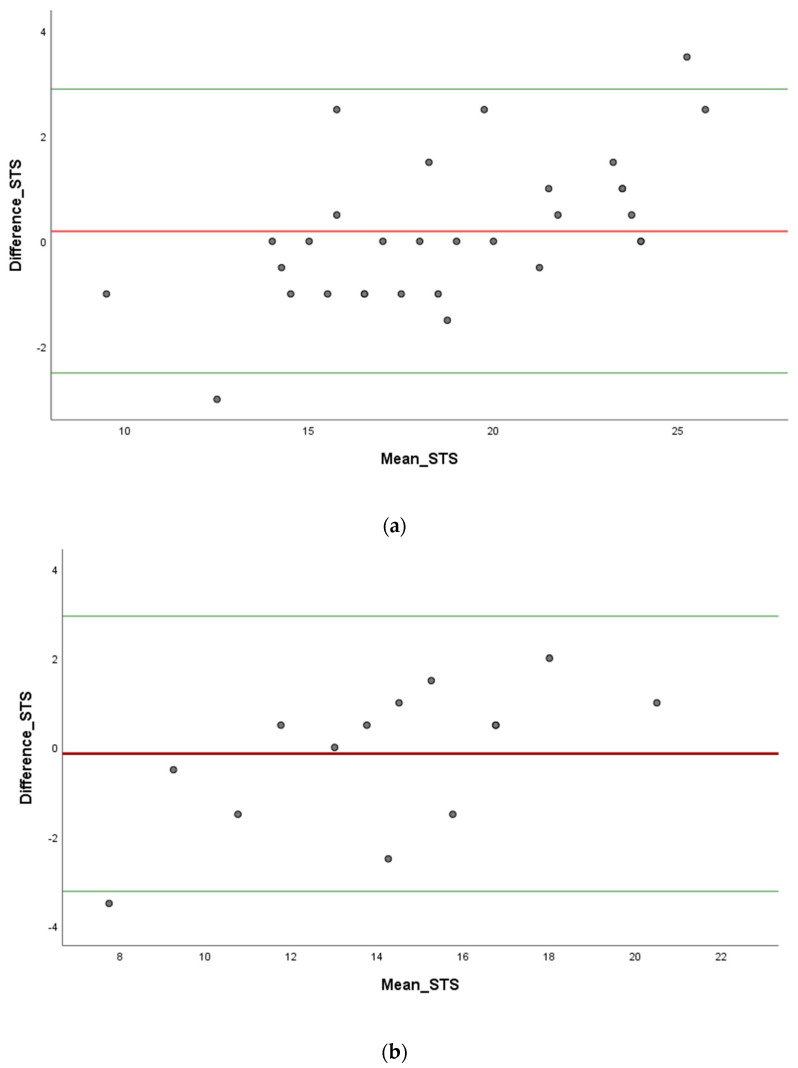
Bland–Altman plots for sit to stand (STS) test. (**a**) Healthy young adults. (**b**) Chronic disease people.

**Figure 6:**
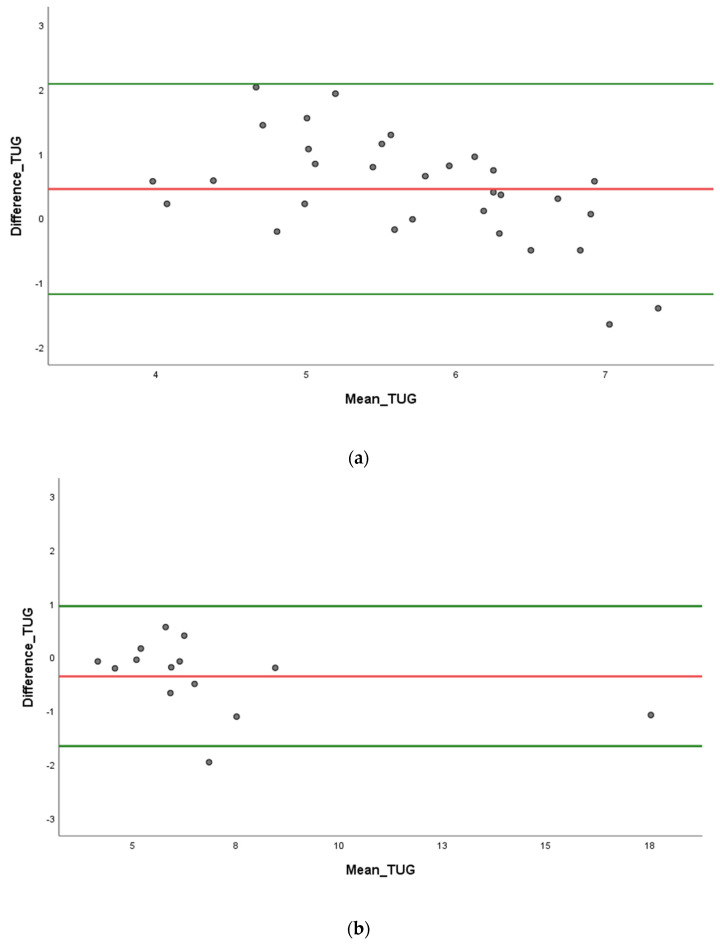
Bland–Altman plots for timed up and go (TUG) test. (**a**) Healthy young adults. (**b**) Chronic disease people.

We used a Bland–Altman graphical approach to check the assumptions of normality of differences between two measures. We quantified agreement between two quantitative measurements by constructing limits of agreement. These statistical limits are calculated by using the mean and the standard deviations of the differences between two measurements. Inter-rater reliability parameters are illustrated by Bland–Altman plots in Figure 5 and Figure 6.

We found a high concordance with proportional bias in chronic disease people during STS (Figure 5b) and TUG (Figure 6b) tests. On the one hand, we found a high concordance with proportional bias in healthy young adults for the STS test (Figure 5a). On the other hand, as shown in Figure 6a, we found the lowest concordance with non-proportional bias in young adults during the TUG test. 

## 4. Discussion

This study evaluated our personal wrist wearable device’s reliability and validity in healthy young adults and chronic disease patients. It compared the output from the custom device to the measure recorded by an investigator for STS and TUG tests. The findings indicate that the custom device provides a valid outcome of a number of STS trials as well as the time for performing the TUG test with high intraclass correlation coefficients compared to estimations observed by the investigator.

Analyses of the relationships between the number of STS provided by the custom device and by observed measures in chronic disease people (r = 0.94) and healthy young adults (r = 0.96) showed excellent and significative correlation (<0.001). The STS intraclass correlation coefficients were 0.90 in chronic disease people and 0.95 in healthy adults. These coefficients are high enough to conclude that our device provides reliable measurements in the STS test using an ICM20948 IMU sensor and suitable processing thanks to the developed algorithms.

Cobo et al. used an ultrasound-based sensor to assess STS and they found a similar result in young adults (with an ICC = 0.96), and in the elderly group (with an ICC = 0.89) compared to our device [20]. Their device comprises an ultrasound sensor, a web board, and a Bluetooth module. In contrast to our system, their device required a special chair with more sensors and owners have noted that the device is difficult for chronic disease people to use alone.

Despite the high sampling rate of our custom device, it slightly underestimates the results of STS and TUG tests in the young adults group. We observed better performance in the healthy youth group (mean STS = 18.9, mean TUG = 5.9 s) compared to the chronic disease group (mean STS = 14.5, mean TUG = 6.7 s), which confirms an overall functional decrease and certain mobility constraints in the chronic disease group. Their movements become less precise, and, during the experimentation, they might have moved their wrist less prominently than healthy young adults. There is probably an age effect, as people with chronic diseases are older and therefore more likely to have poor physical activity performance compared to young adults [18]. A systematic review of the literature on previous studies highlighted this limitation related to the use of IMU for the assessment of chronic disease and elderly people [18]. The main objective of our work is to adapt our device as much as possible to chronic disease people, so the algorithms are more specific to their functioning. In addition, during the evaluations, the chronic disease patients performed the tests with more applications because they are familiar with them, unlike young adults.

In a recent study, Fudickar et al. used a sensor placed on the chest of patients. They also observed a significant correlation (r = 0.73) between the results provided by the device and the stop-watch during the TUG. On the other hand, they noted difficulty with chronic diseases people wearing the sensor and handling it [31], which could indicate age-related (mean, 79.41 years) limitations such as reduced impaired motor and sensory [32,33]. Although the strap was already lengthened in their study, some participants, especially those with higher body weight, still felt it was too narrow and should be lengthened again. In our study, no such difficulties were reported, so the participants found the use of our device very useful.

### 4.1. Accuracy

In this study, the most accurate estimations were obtained using the custom device for STS tests in young adults (RMSE = 7.22%), chronic disease people (RMSE = 10.83%) but also for TUG test in chronic disease people (RMSE = 11.02%). The custom device showed lower accuracy for the TUG test in healthy adults (RMSE = 15.75%). This lower precision could be explained by the fact that the algorithm was adapted for people with chronic diseases, but we also used it for healthy people. 

The findings of our study showed that mean Handheld Stopwatch (HHS) times during TUG was 0.08 s faster than our custom device in the chronic disease group. These values are in close agreement with Hetzler et al., who have reported HHS times to be 0.07 s faster than electronic timing in 200-m sprint [34]. However, in the healthy group, we found that the mean HHS during TUG was 0.13 s lower than our custom device. In another study, Moore et al., have reported HHS times to be 0.08 s slower than electronic times [35]. The authors attribute these conflicting results to differences in starting procedures used by HHS operators. It is possible that factors associated with the study itself, including the speed of the runners and the starting protocol employed, may have affected the accuracy of HHS times relative to electronic times [34]. It is possible that faster participants (healthy adults) may be more difficult to time accurately using HHS during the TUG, thus increasing the possibility of timing errors.

Additionally, during the TUG test, some healthy adults had to hold their wrists still for a while to look at the screen to ensure the watch was recording activity during the TUG test. They may thus have degraded the reliability. However, we did not collect data to support this hypothesis. We found no other work evaluating the accuracy and error rate following the use of a wrist wearable IMU device to assess STS and TUG in chronic disease people. 

Our device is worn on the wrist and remains less bulky compared with existing devices. Complex devices show a low utilization rate in contrast to wrist-worn devices that are easy to wear unaided by chronic disease people. In future studies, we need to evaluate the acceptability of our wrist-worn IMU device. The latter has proven to be accurate, but the ease of use and user experience of chronic disease people remains unknown.

### 4.2. Limitations

We are aware that the study includes some limitations. The main limitation is the method used to assess the accuracy of the results provided by our custom device. There is no consensus on the acceptable error rate (for MB and RMSE). The authors recommend having the minimum error possible, the error rate varies between 5 and 15% [36]. 

Furthermore, it is important to remember that, although the groups were divided into “healthy” and “chronically disease” subjects, no intra-group separation was made, which would necessitate taking into consideration the characteristics and differences between men and women, and the specific diseases of the participants when interpreting the results.

### 4.3. Strengths of the Study

Indeed, there are expensive, complex and cumbersome devices to assess patients’ balance, as opposed to our custom device. To our knowledge, to date, no study has investigated the reliability of a wearable wrist sensor to assess patients’ condition using physical fitness tests. These findings allow us to evaluate chronic disease patients’ physical fitness at a distance using the IMU devices with our algorithms. This wearable device will be integrated into a web platform to allow healthcare professionals to remotely monitor the fitness of chronic disease patients and to guide them in their effort training with adapted exercises. The excellent correlations between our personal measures and observed measures indicate a reliable measure. Our system produced valid results but also an acceptable accuracy with reference to the existing literature.

## 5. Conclusions

For both young healthy adults and chronic disease people, this integrated wearable device provides valid numbers of STS and seconds in the TUG test with acceptable accuracy. Inter-device reliability is generally good for STS and TUG testing in healthy young adults and people with chronic diseases. The treatments and algorithms developed from the raw data are therefore suitable for people with chronic diseases.

These findings make this wrist-worn device particularly useful for remotely monitoring progress in mobility and strength in patients with chronic conditions. IMU devices can then be easily integrated into a web-based platform so that therapists can automate the physical fitness assessments in order to follow chronic disease patients and improve their life condition. Future studies will be necessary to consolidate the results observed in this first study by working on a larger population. Based on this study, it would be important to conduct future work to integrate other algorithms to assess other physical skills, such as endurance and static balance.

## Figures and Tables

**Table 1:** Main characteristics of the device.

Characteristics	Value
IMU 9-axis sensor	ICM20948
Microcontroller processing unit	STM32WB55RG
On chip memory (program + data)	1 Mbyte
DAC resolution	12 bits
Sampling frequency	50 Hz
Connectivity	BLE and USB
Power	Lithium ion battery 560 mAh 3.7 V

**Table 2:** Demographic characteristics of the population.

Characteristic	Healthy Adults, n = 31	Chronic Disease, n = 14
Sex		
Female	15 (48%)	9 (64%)
Male	16 (52%)	5 (36%)
Weight (Kg)	70.0 [12]	82.0 [10]
Height (m)	1.7 ± 0.1	1.6 ± 0.1
STS observed (n)	18.9 ± 4.5	14.1 ± 4.0
STS sensor (n)	18.7 ± 3.8	14.2 ± 3.0
TUG observed (s)	5.9 ± 0.8	6.7 ± 3.1
TUG Sensor (s)	5.5 ± 1.2	7.0 ± 3.4

The values are expressed in Median [IQR]: Interquartile range, Mean ± Standard deviation); Kg: Kilogram; m: meter; STS: Sit to stand test; n: number; TUG: Timed Up and Go test; s: second.

**Table 3:** Validity parameters of the results estimated by wearable custom device versus results observed by examiner.

Measures	r	*p*-Value		ICC	95% CI	*p*-Value
STS group A	0.96	<0.001		0.95	0.9, 0.97	<0.001
STS group B	0.94	<0.001		0.90	0.74, 0.97	<0.001
TUG group A	0.80	<0.001		0.75	0.43, 0.79	<0.001
TUG group B	0.87	<0.001		0.98	0.91, 0.99	<0.001

STS = Sit-to-stand test, TUG = Timed Up and Go test; group A = healthy group; group B = chronic disease group; r = Spearman correlation coefficient; *p*-value = statistical significance; ICC = Intraclass correlation coefficient; CI = Confidence Intervals.

**Table 4:** Inter-rater reliability parameters of the results estimated by wearable custom device versus results observed by examiner.

Measures	Mean Bias	Percentage Difference (%)	95% LoA Down	95% LoA Up	RMSE	Percentage RMSE (%)
STS group A	0.19	0.08	−2.5	2.89	1.37	7.22
STS group B	−0.14	−3.21	−3.23	2.94	1.52	10.83
TUG group A	0.45	9.24	−1.18	2.08	0.94	15.75
TUG group B	−0.36	−4.56	−1.66	0.95	0.74	11.02

STS = Sit-to-stand test (expressed in number), TUG = Timed Up and Go test (expressed in second); group A = healthy group; group B = chronic disease group; Percentage Difference = mean bias expressed in percentage of results measured and estimated by device; 95% LoA = limits of agreement of Bland–Altman analysis; RMSE = root-mean-square error; Percentage RMSE = RMSE expressed in percentage of result observed by examiner.

## Data Availability

In accordance with the RGPD law in effect in France and the requirements of the ethics committee, the data from this study are stored on a secure server. The datasets generated for this study are available on request to the corresponding authors.
